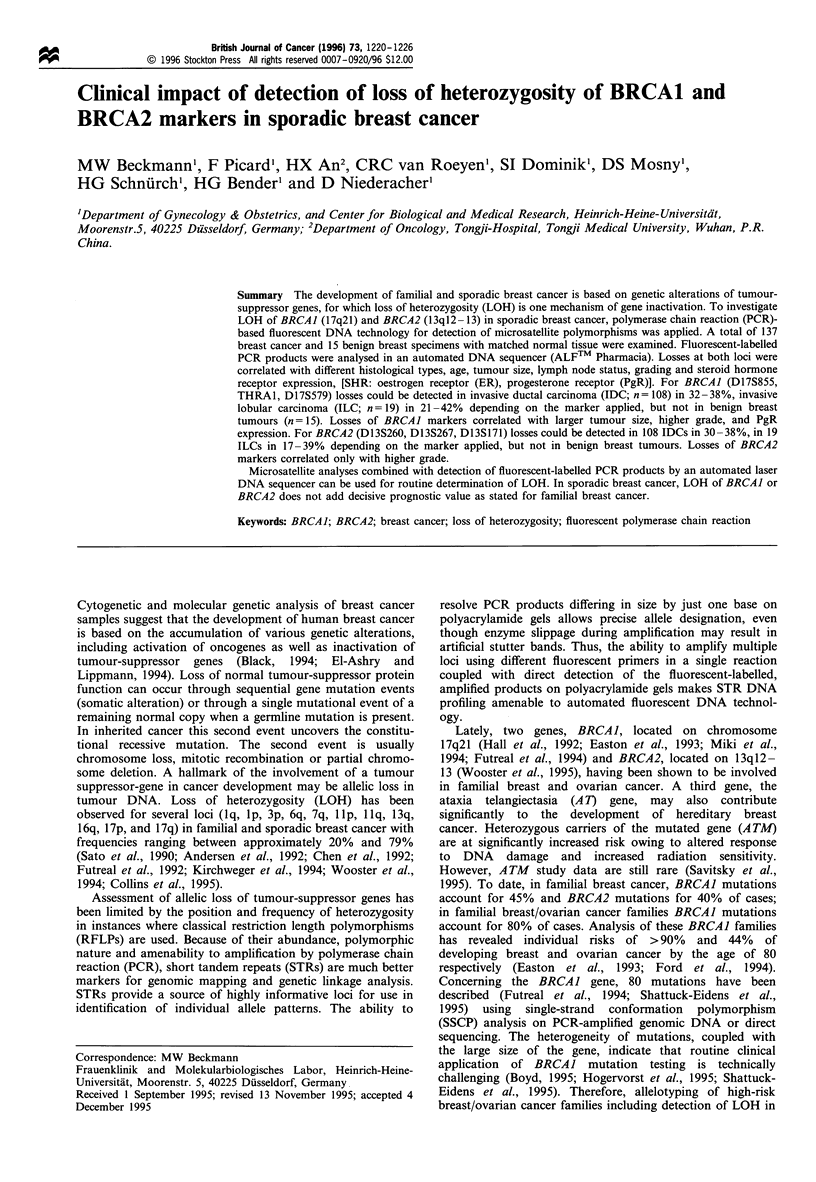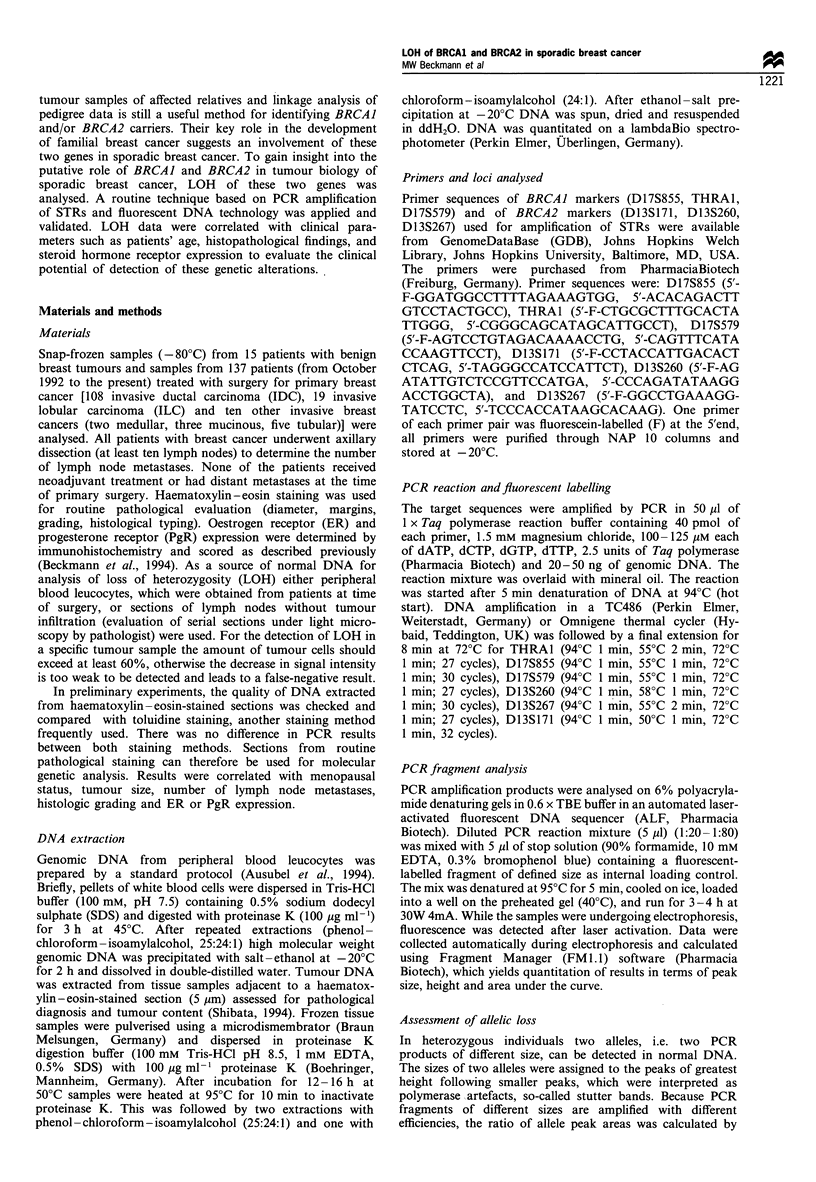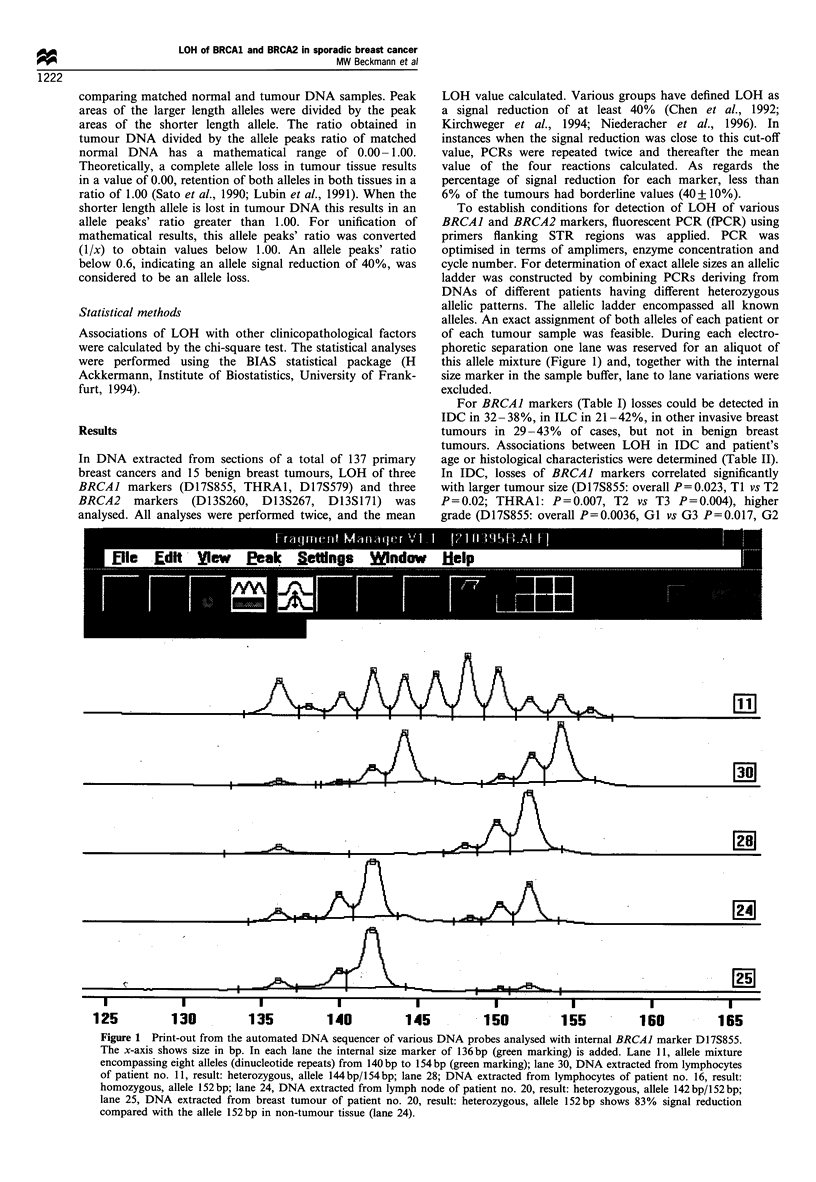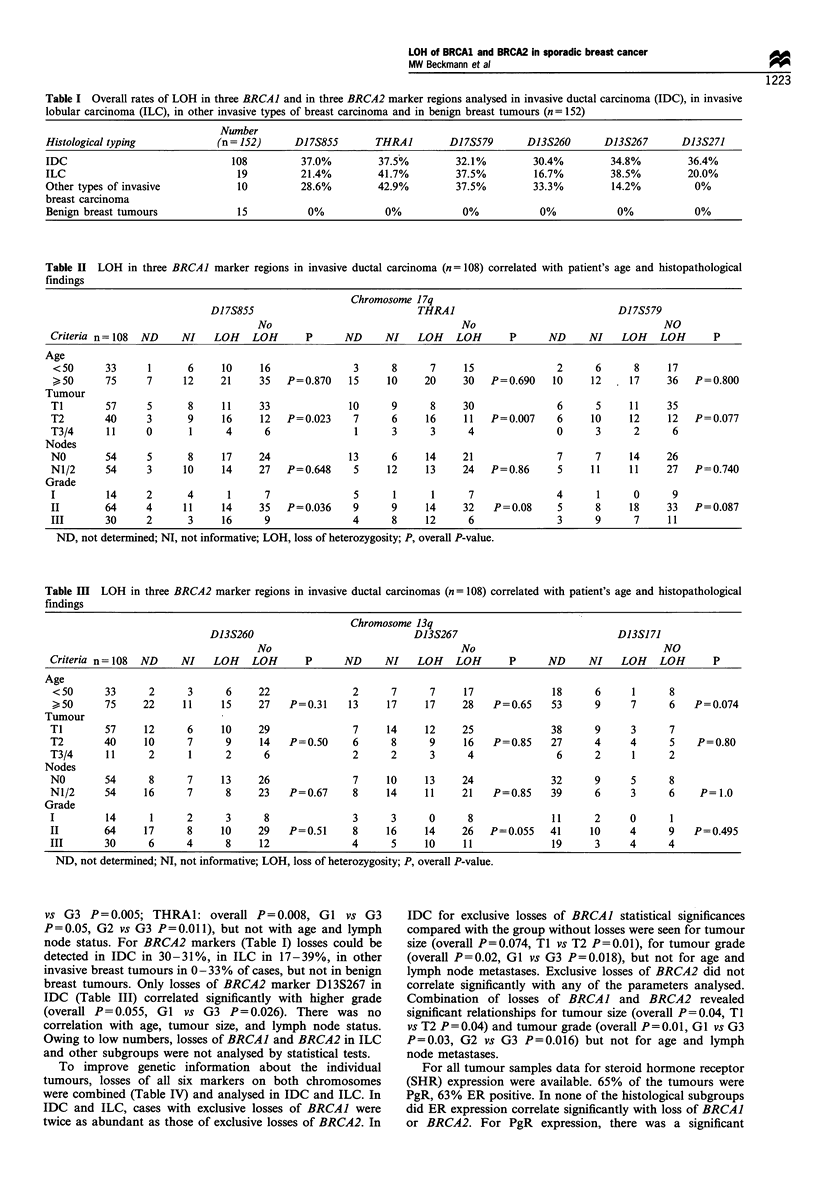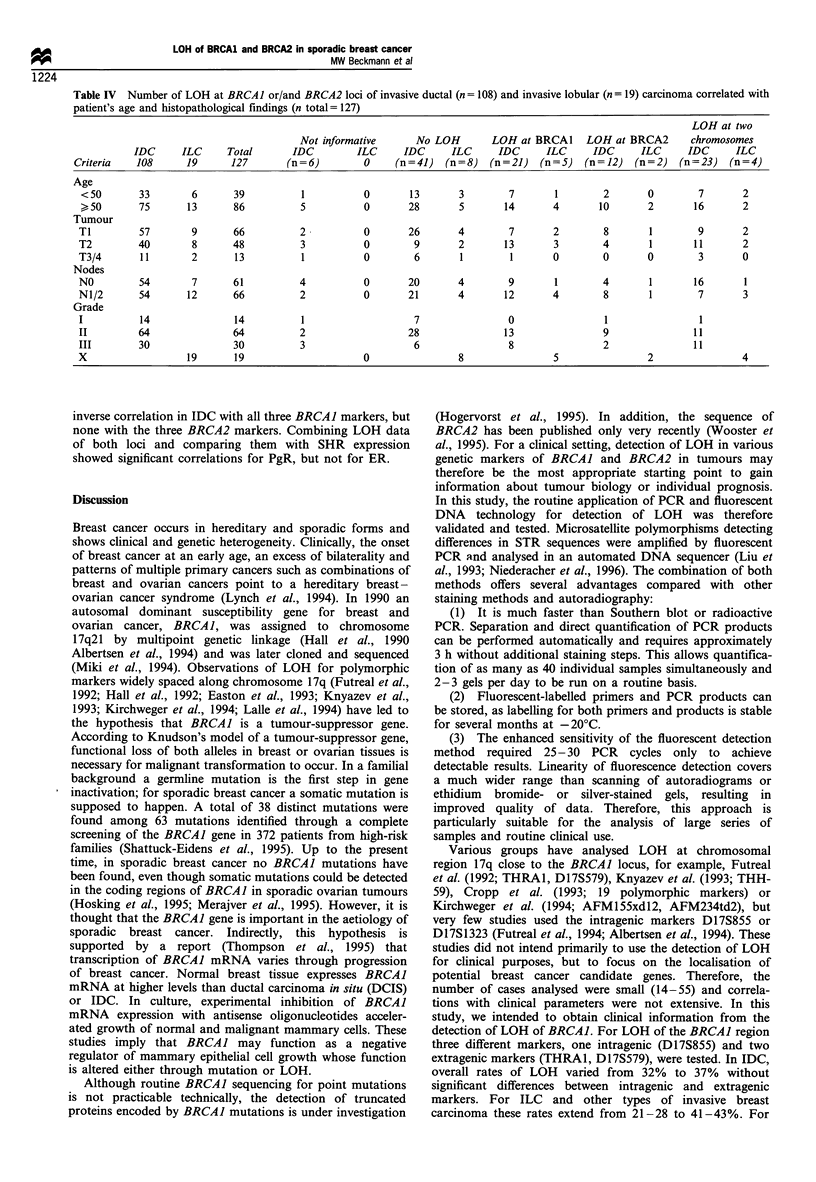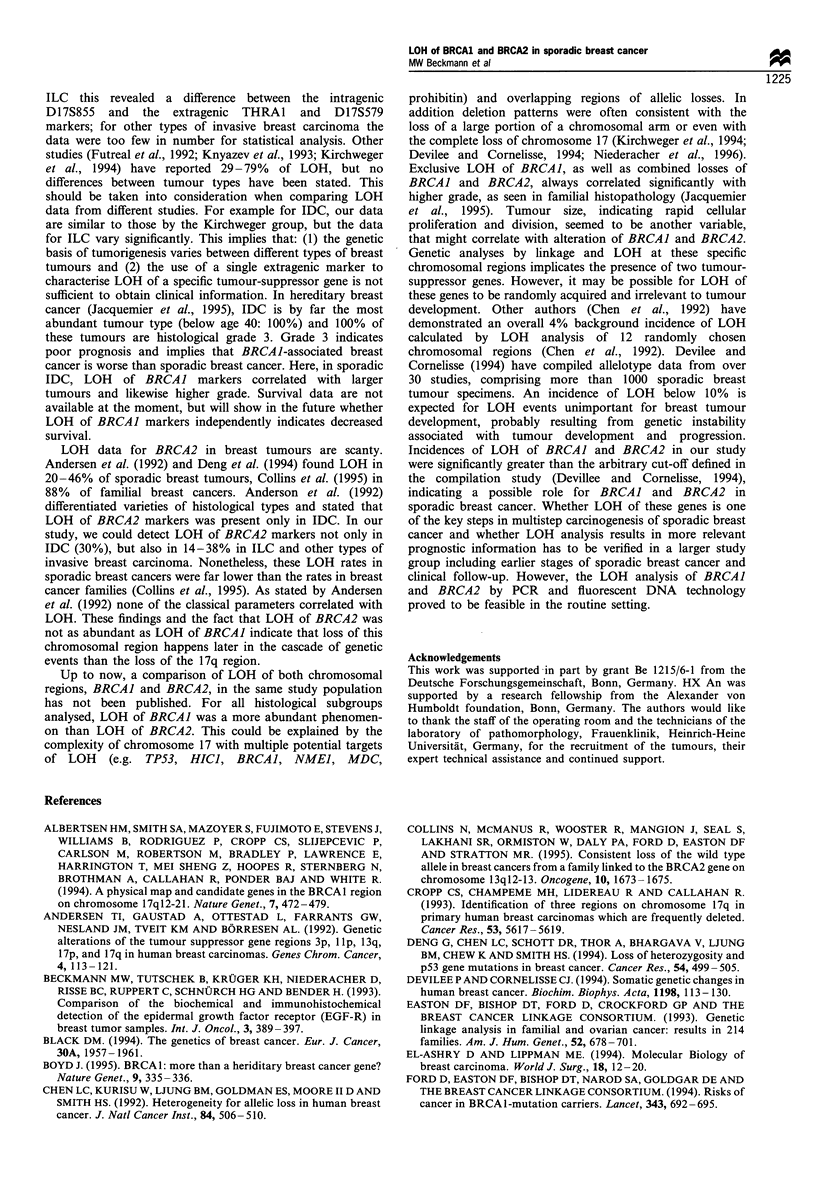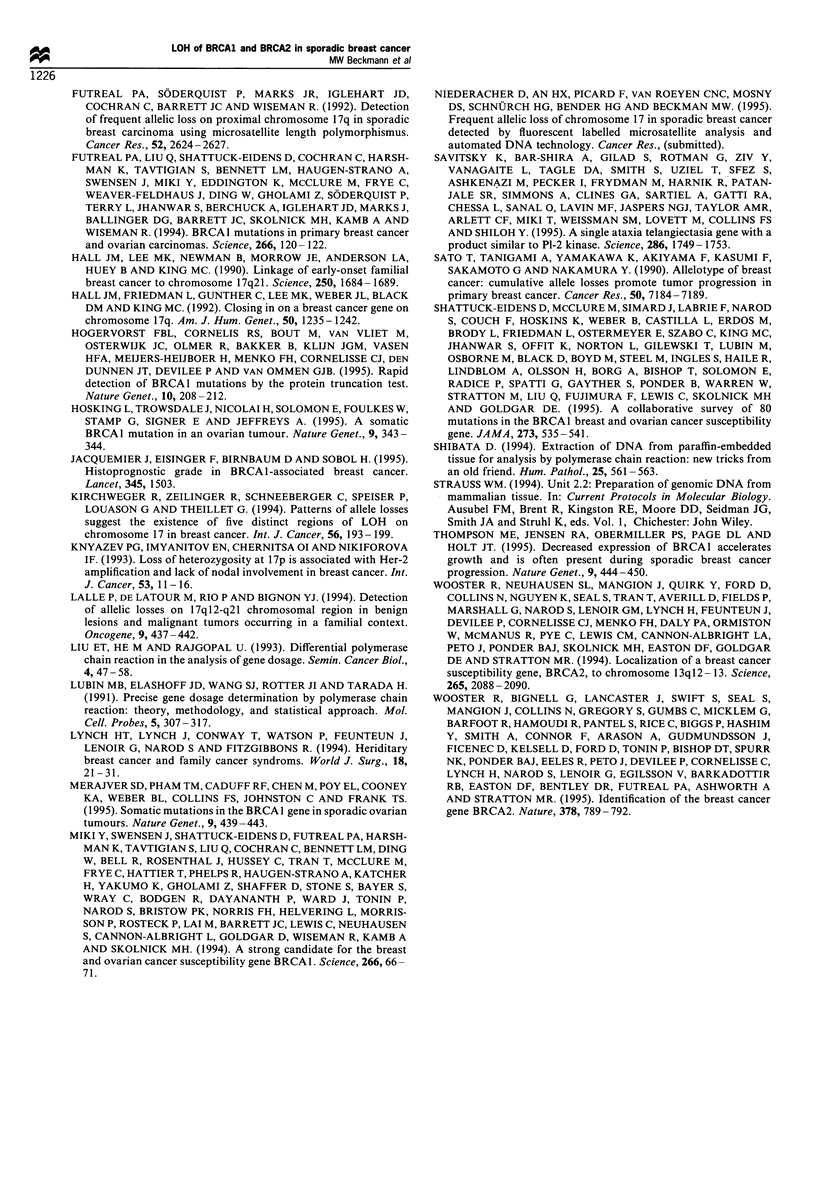# Clinical impact of detection of loss of heterozygosity of BRCA1 and BRCA2 markers in sporadic breast cancer.

**DOI:** 10.1038/bjc.1996.234

**Published:** 1996-05

**Authors:** M. W. Beckmann, F. Picard, H. X. An, C. R. van Roeyen, S. I. Dominik, D. S. Mosny, H. G. Schnürch, H. G. Bender, D. Niederacher

**Affiliations:** Department of Gynecology & Obstetrics, Heinrich-Heine-Universität, Düsseldorf, Germany.

## Abstract

**Images:**


British Journal of Cancer (1996) 73, 1220-1226
© 1996 Stockton Press All rights reserved 0007-0920/96 $12.00

### Clinical impact of detection of loss of heterozygosity of BRCA1 and BRCA2 markers in sporadic breast cancer

MW Beckmann[1], F Picard[1], HX An[2], CRC van Roeyen[1], SI Dominik[1], DS Mosny[1], HG Schnürch[1], HG Bender[1] and D Niederacher[1]

[1]*Department of Gynecology & Obstetrics, and Center for Biological and Medical Research, Heinrich-Heine-Universität, Moorenstr.5, 40225 Düsseldorf, Germany;* [2]*Department of Oncology, Tongji-Hospital, Tongji Medical University, Wuhan, P.R. China.*

**Summary** The development of familial and sporadic breast cancer is based on genetic alterations of tumour-suppressor genes, for which loss of heterozygosity (LOH) is one mechanism of gene inactivation. To investigate LOH of *BRCA1* (17q21) and *BRCA2* (13q12-13) in sporadic breast cancer, polymerase chain reaction (PCR)-based fluorescent DNA technology for detection of microsatellite polymorphisms was applied. A total of 137 breast cancer and 15 benign breast specimens with matched normal tissue were examined. Fluorescent-labelled PCR products were analysed in an automated DNA sequencer (ALF$^{TM}$ Pharmacia). Losses at both loci were correlated with different histological types, age, tumour size, lymph node status, grading and steroid hormone receptor expression, [SHR: oestrogen receptor (ER), progesterone receptor (PgR)]. For *BRCA1* (D17S855, THRA1, D17S579) losses could be detected in invasive ductal carcinoma (IDC; $n=108$) in 32-38%, invasive lobular carcinoma (ILC; $n=19$) in 21-42% depending on the marker applied, but not in benign breast tumours ($n=15$). Losses of *BRCA1* markers correlated with larger tumour size, higher grade, and PgR expression. For *BRCA2* (D13S260, D13S267, D13S171) losses could be detected in 108 IDCs in 30-38%, in 19 ILCs in 17-39% depending on the marker applied, but not in benign breast tumours. Losses of *BRCA2* markers correlated only with higher grade.

Microsatellite analyses combined with detection of fluorescent-labelled PCR products by an automated laser DNA sequencer can be used for routine determination of LOH. In sporadic breast cancer, LOH of *BRCA1* or *BRCA2* does not add decisive prognostic value as stated for familial breast cancer.

**Keywords:** *BRCA1*; *BRCA2*; breast cancer; loss of heterozygosity; fluorescent polymerase chain reaction

Cytogenetic and molecular genetic analysis of breast cancer samples suggest that the development of human breast cancer is based on the accumulation of various genetic alterations, including activation of oncogenes as well as inactivation of tumour-suppressor genes (Black, 1994; El-Ashry and Lippmann, 1994). Loss of normal tumour-suppressor protein function can occur through sequential gene mutation events (somatic alteration) or through a single mutational event of a remaining normal copy when a germline mutation is present. In inherited cancer this second event uncovers the constitutional recessive mutation. The second event is usually chromosome loss, mitotic recombination or partial chromosome deletion. A hallmark of the involvement of a tumour suppressor-gene in cancer development may be allelic loss in tumour DNA. Loss of heterozygosity (LOH) has been observed for several loci (1q, 1p, 3p, 6q, 7q, 11p, 11q, 13q, 16q, 17p, and 17q) in familial and sporadic breast cancer with frequencies ranging between approximately 20% and 79% (Sato *et al.*, 1990; Andersen *et al.*, 1992; Chen *et al.*, 1992; Futreal *et al.*, 1992; Kirchweger *et al.*, 1994; Wooster *et al.*, 1994; Collins *et al.*, 1995).

Assessment of allelic loss of tumour-suppressor genes has been limited by the position and frequency of heterozygosity in instances where classical restriction length polymorphisms (RFLPs) are used. Because of their abundance, polymorphic nature and amenability to amplification by polymerase chain reaction (PCR), short tandem repeats (STRs) are much better markers for genomic mapping and genetic linkage analysis. STRs provide a source of highly informative loci for use in identification of individual allele patterns. The ability to resolve PCR products differing in size by just one base on polyacrylamide gels allows precise allele designation, even though enzyme slippage during amplification may result in artificial stutter bands. Thus, the ability to amplify multiple loci using different fluorescent primers in a single reaction coupled with direct detection of the fluorescent-labelled, amplified products on polyacrylamide gels makes STR DNA profiling amenable to automated fluorescent DNA technology.

Lately, two genes, *BRCA1*, located on chromosome 17q21 (Hall *et al.*, 1992; Easton *et al.*, 1993; Miki *et al.*, 1994; Futreal *et al.*, 1994) and *BRCA2*, located on 13q12-13 (Wooster *et al.*, 1995), having been shown to be involved in familial breast and ovarian cancer. A third gene, the ataxia telangiectasia (*AT*) gene, may also contribute significantly to the development of hereditary breast cancer. Heterozygous carriers of the mutated gene (*ATM*) are at significantly increased risk owing to altered response to DNA damage and increased radiation sensitivity. However, *ATM* study data are still rare (Savitsky *et al.*, 1995). To date, in familial breast cancer, *BRCA1* mutations account for 45% and *BRCA2* mutations for 40% of cases; in familial breast/ovarian cancer families *BRCA1* mutations account for 80% of cases. Analysis of these *BRCA1* families has revealed individual risks of >90% and 44% of developing breast and ovarian cancer by the age of 80 respectively (Easton *et al.*, 1993; Ford *et al.*, 1994). Concerning the *BRCA1* gene, 80 mutations have been described (Futreal *et al.*, 1994; Shattuck-Eidens *et al.*, 1995) using single-strand conformation polymorphism (SSCP) analysis on PCR-amplified genomic DNA or direct sequencing. The heterogeneity of mutations, coupled with the large size of the gene, indicate that routine clinical application of *BRCA1* mutation testing is technically challenging (Boyd, 1995; Hogervorst *et al.*, 1995; Shattuck-Eidens *et al.*, 1995). Therefore, allelotyping of high-risk breast/ovarian cancer families including detection of LOH in

Correspondence: MW Beckmann
Frauenklinik and Molekularbiologisches Labor, Heinrich-Heine-Universität, Moorenstr. 5, 40225 Düsseldorf, Germany
Received 1 September 1995; revised 13 November 1995; accepted 4 December 1995

tumour samples of affected relatives and linkage analysis of pedigree data is still a useful method for identifying *BRCA1* and/or *BRCA2* carriers. Their key role in the development of familial breast cancer suggests an involvement of these two genes in sporadic breast cancer. To gain insight into the putative role of *BRCA1* and *BRCA2* in tumour biology of sporadic breast cancer, LOH of these two genes was analysed. A routine technique based on PCR amplification of STRs and fluorescent DNA technology was applied and validated. LOH data were correlated with clinical parameters such as patients' age, histopathological findings, and steroid hormone receptor expression to evaluate the clinical potential of detection of these genetic alterations.

### Materials and methods

### *Materials*

Snap-frozen samples (−80°C) from 15 patients with benign breast tumours and samples from 137 patients (from October 1992 to the present) treated with surgery for primary breast cancer [108 invasive ductal carcinoma (IDC), 19 invasive lobular carcinoma (ILC) and ten other invasive breast cancers (two medullar, three mucinous, five tubular)] were analysed. All patients with breast cancer underwent axillary dissection (at least ten lymph nodes) to determine the number of lymph node metastases. None of the patients received neoadjuvant treatment or had distant metastases at the time of primary surgery. Haematoxylin–eosin staining was used for routine pathological evaluation (diameter, margins, grading, histological typing). Oestrogen receptor (ER) and progesterone receptor (PgR) expression were determined by immunohistochemistry and scored as described previously (Beckmann *et al.*, 1994). As a source of normal DNA for analysis of loss of heterozygosity (LOH) either peripheral blood leucocytes, which were obtained from patients at time of surgery, or sections of lymph nodes without tumour infiltration (evaluation of serial sections under light microscopy by pathologist) were used. For the detection of LOH in a specific tumour sample the amount of tumour cells should exceed at least 60%, otherwise the decrease in signal intensity is too weak to be detected and leads to a false-negative result.

In preliminary experiments, the quality of DNA extracted from haematoxylin–eosin-stained sections was checked and compared with toluidine staining, another staining method frequently used. There was no difference in PCR results between both staining methods. Sections from routine pathological staining can therefore be used for molecular genetic analysis. Results were correlated with menopausal status, tumour size, number of lymph node metastases, histologic grading and ER or PgR expression.

### *DNA extraction*

Genomic DNA from peripheral blood leucocytes was prepared by a standard protocol (Ausubel *et al.*, 1994). Briefly, pellets of white blood cells were dispersed in Tris-HCl buffer (100 mM, pH 7.5) containing 0.5% sodium dodecyl sulphate (SDS) and digested with proteinase K (100 $\mu g\ ml^{-1}$) for 3 h at 45°C. After repeated extractions (phenol–chloroform–isoamylalcohol, 25:24:1) high molecular weight genomic DNA was precipitated with salt–ethanol at −20°C for 2 h and dissolved in double-distilled water. Tumour DNA was extracted from tissue samples adjacent to a haematoxylin–eosin-stained section (5 $\mu m$) assessed for pathological diagnosis and tumour content (Shibata, 1994). Frozen tissue samples were pulverised using a microdismembrator (Braun Melsungen, Germany) and dispersed in proteinase K digestion buffer (100 mM Tris-HCl pH 8.5, 1 mM EDTA, 0.5% SDS) with 100 $\mu g\ ml^{-1}$ proteinase K (Boehringer, Mannheim, Germany). After incubation for 12–16 h at 50°C samples were heated at 95°C for 10 min to inactivate proteinase K. This was followed by two extractions with phenol–chloroform–isoamylalcohol (25:24:1) and one with chloroform–isoamylalcohol (24:1). After ethanol–salt precipitation at −20°C DNA was spun, dried and resuspended in $ddH_2O$. DNA was quantitated on a lambdaBio spectrophotometer (Perkin Elmer, Überlingen, Germany).

### *Primers and loci analysed*

Primer sequences of *BRCA1* markers (D17S855, THRA1, D17S579) and of *BRCA2* markers (D13S171, D13S260, D13S267) used for amplification of STRs were available from GenomeDataBase (GDB), Johns Hopkins Welch Library, Johns Hopkins University, Baltimore, MD, USA. The primers were purchased from PharmaciaBiotech (Freiburg, Germany). Primer sequences were: D17S855 (5′-F-GGATGGCCTTTTAGAAAGTGG, 5′-ACACAGACTT GTCCTACTGCC), THRA1 (5′-F-CTGCGCTTTGCACTA TTGGG, 5′-CGGGCAGCATAGCATTGCCT), D17S579 (5′-F-AGTCCTGTAGACAAAACCTG, 5′-CAGTTTCATA CCAAGTTCCT), D13S171 (5′-F-CCTACCATTGACACT CTCAG, 5′-TAGGGCCATCCATTCT), D13S260 (5′-F-AG ATATTGTCTCCGTTCCATGA, 5′-CCCAGATATAAGG ACCTGGCTA), and D13S267 (5′-F-GGCCTGAAAGG-TATCCTC, 5′-TCCCACCATAAGCACAAG). One primer of each primer pair was fluorescein-labelled (F) at the 5′end, all primers were purified through NAP 10 columns and stored at −20°C.

### *PCR reaction and fluorescent labelling*

The target sequences were amplified by PCR in 50 $\mu l$ of 1 × *Taq* polymerase reaction buffer containing 40 pmol of each primer, 1.5 mM magnesium chloride, 100–125 $\mu M$ each of dATP, dCTP, dGTP, dTTP, 2.5 units of *Taq* polymerase (Pharmacia Biotech) and 20–50 ng of genomic DNA. The reaction mixture was overlaid with mineral oil. The reaction was started after 5 min denaturation of DNA at 94°C (hot start). DNA amplification in a TC486 (Perkin Elmer, Weiterstadt, Germany) or Omnigene thermal cycler (Hybaid, Teddington, UK) was followed by a final extension for 8 min at 72°C for THRA1 (94°C 1 min, 55°C 2 min, 72°C 1 min; 27 cycles), D17S855 (94°C 1 min, 55°C 1 min, 72°C 1 min; 30 cycles), D17S579 (94°C 1 min, 55°C 1 min, 72°C 1 min; 27 cycles), D13S260 (94°C 1 min, 58°C 1 min, 72°C 1 min; 30 cycles), D13S267 (94°C 1 min, 55°C 2 min, 72°C 1 min; 27 cycles), D13S171 (94°C 1 min, 50°C 1 min, 72°C 1 min, 32 cycles).

### *PCR fragment analysis*

PCR amplification products were analysed on 6% polyacrylamide denaturing gels in 0.6 × TBE buffer in an automated laser-activated fluorescent DNA sequencer (ALF, Pharmacia Biotech). Diluted PCR reaction mixture (5 $\mu l$) (1:20–1:80) was mixed with 5 $\mu l$ of stop solution (90% formamide, 10 mM EDTA, 0.3% bromophenol blue) containing a fluorescent-labelled fragment of defined size as internal loading control. The mix was denatured at 95°C for 5 min, cooled on ice, loaded into a well on the preheated gel (40°C), and run for 3–4 h at 30W 4mA. While the samples were undergoing electrophoresis, fluorescence was detected after laser activation. Data were collected automatically during electrophoresis and calculated using Fragment Manager (FM1.1) software (Pharmacia Biotech), which yields quantitation of results in terms of peak size, height and area under the curve.

### *Assessment of allelic loss*

In heterozygous individuals two alleles, i.e. two PCR products of different size, can be detected in normal DNA. The sizes of two alleles were assigned to the peaks of greatest height following smaller peaks, which were interpreted as polymerase artefacts, so-called stutter bands. Because PCR fragments of different sizes are amplified with different efficiencies, the ratio of allele peak areas was calculated by comparing matched normal and tumour DNA samples. Peak areas of the larger length alleles were divided by the peak areas of the shorter length allele. The ratio obtained in tumour DNA divided by the allele peaks ratio of matched normal DNA has a mathematical range of 0.00–1.00. Theoretically, a complete allele loss in tumour tissue results in a value of 0.00, retention of both alleles in both tissues in a ratio of 1.00 (Sato *et al*., 1990; Lubin *et al*., 1991). When the shorter length allele is lost in tumour DNA this results in an allele peaks' ratio greater than 1.00. For unification of mathematical results, this allele peaks' ratio was converted ($1/x$) to obtain values below 1.00. An allele peaks' ratio below 0.6, indicating an allele signal reduction of 40%, was considered to be an allele loss.

### *Statistical methods*

Associations of LOH with other clinicopathological factors were calculated by the chi-square test. The statistical analyses were performed using the BIAS statistical package (H Ackkermann, Institute of Biostatistics, University of Frankfurt, 1994).

### Results

In DNA extracted from sections of a total of 137 primary breast cancers and 15 benign breast tumours, LOH of three *BRCA1* markers (D17S855, THRA1, D17S579) and three *BRCA2* markers (D13S260, D13S267, D13S171) was analysed. All analyses were performed twice, and the mean LOH value calculated. Various groups have defined LOH as a signal reduction of at least 40% (Chen *et al*., 1992; Kirchweger *et al*., 1994; Niederacher *et al*., 1996). In instances when the signal reduction was close to this cut-off value, PCRs were repeated twice and thereafter the mean value of the four reactions calculated. As regards the percentage of signal reduction for each marker, less than 6% of the tumours had borderline values ($40 \pm 10\%$).

To establish conditions for detection of LOH of various *BRCA1* and *BRCA2* markers, fluorescent PCR (fPCR) using primers flanking STR regions was applied. PCR was optimised in terms of amplimers, enzyme concentration and cycle number. For determination of exact allele sizes an allelic ladder was constructed by combining PCRs deriving from DNAs of different patients having different heterozygous allelic patterns. The allelic ladder encompassed all known alleles. An exact assignment of both alleles of each patient or of each tumour sample was feasible. During each electrophoretic separation one lane was reserved for an aliquot of this allele mixture (Figure 1) and, together with the internal size marker in the sample buffer, lane to lane variations were excluded.

For *BRCA1* markers (Table I) losses could be detected in IDC in 32–38%, in ILC in 21–42%, in other invasive breast tumours in 29–43% of cases, but not in benign breast tumours. Associations between LOH in IDC and patient's age or histological characteristics were determined (Table II). In IDC, losses of *BRCA1* markers correlated significantly with larger tumour size (D17S855: overall $P=0.023$, T1 *vs* T2 $P=0.02$; THRA1: $P=0.007$, T2 *vs* T3 $P=0.004$), higher grade (D17S855: overall $P=0.0036$, G1 *vs* G3 $P=0.017$, G2

**Figure 1** Print-out from the automated DNA sequencer of various DNA probes analysed with internal *BRCA1* marker D17S855. The *x*-axis shows size in bp. In each lane the internal size marker of 136 bp (green marking) is added. Lane 11, allele mixture encompassing eight alleles (dinucleotide repeats) from 140 bp to 154 bp (green marking); lane 30, DNA extracted from lymphocytes of patient no. 11, result: heterozygous, allele 144 bp/154 bp; lane 28; DNA extracted from lymphocytes of patient no. 16, result: homozygous, allele 152 bp; lane 24, DNA extracted from lymph node of patient no. 20, result: heterozygous, allele 142 bp/152 bp; lane 25, DNA extracted from breast tumour of patient no. 20, result: heterozygous, allele 152 bp shows 83% signal reduction compared with the allele 152 bp in non-tumour tissue (lane 24).

**Table I** Overall rates of LOH in three *BRCA1* and in three *BRCA2* marker regions analysed in invasive ductal carcinoma (IDC), in invasive lobular carcinoma (ILC), in other invasive types of breast carcinoma and in benign breast tumours ($n=152$)

| *Histological typing* | *Number (n=152)* | *D17S855* | *THRA1* | *D17S579* | *D13S260* | *D13S267* | *D13S271* |
|---|---|---|---|---|---|---|---|
| IDC | 108 | 37.0% | 37.5% | 32.1% | 30.4% | 34.8% | 36.4% |
| ILC | 19 | 21.4% | 41.7% | 37.5% | 16.7% | 38.5% | 20.0% |
| Other types of invasive breast carcinoma | 10 | 28.6% | 42.9% | 37.5% | 33.3% | 14.2% | 0% |
| Benign breast tumours | 15 | 0% | 0% | 0% | 0% | 0% | 0% |

**Table II** LOH in three *BRCA1* marker regions in invasive ductal carcinoma ($n=108$) correlated with patient's age and histopathological findings

| | | *Chromosome 17q* | | | | | | | | | | | | | | |
|---|---|---|---|---|---|---|---|---|---|---|---|---|---|---|---|---|
| | | *D17S855* | | | | | *THRA1* | | | | | *D17S579* | | | | |
| *Criteria* | n=108 | *ND* | *NI* | *LOH* | *No LOH* | P | *ND* | *NI* | *LOH* | *No LOH* | P | *ND* | *NI* | *LOH* | *NO LOH* | P |
| Age | | | | | | | | | | | | | | | | |
| <50 | 33 | 1 | 6 | 10 | 16 | | 3 | 8 | 7 | 15 | | 2 | 6 | 8 | 17 | |
| ⩾50 | 75 | 7 | 12 | 21 | 35 | $P=0.870$ | 15 | 10 | 20 | 30 | $P=0.690$ | 10 | 12 | 17 | 36 | $P=0.800$ |
| Tumour | | | | | | | | | | | | | | | | |
| T1 | 57 | 5 | 8 | 11 | 33 | | 10 | 9 | 8 | 30 | | 6 | 5 | 11 | 35 | |
| T2 | 40 | 3 | 9 | 16 | 12 | $P=0.023$ | 7 | 6 | 16 | 11 | $P=0.007$ | 6 | 10 | 12 | 12 | $P=0.077$ |
| T3/4 | 11 | 0 | 1 | 4 | 6 | | 1 | 3 | 3 | 4 | | 0 | 3 | 2 | 6 | |
| Nodes | | | | | | | | | | | | | | | | |
| N0 | 54 | 5 | 8 | 17 | 24 | | 13 | 6 | 14 | 21 | | 7 | 7 | 14 | 26 | |
| N1/2 | 54 | 3 | 10 | 14 | 27 | $P=0.648$ | 5 | 12 | 13 | 24 | $P=0.86$ | 5 | 11 | 11 | 27 | $P=0.740$ |
| Grade | | | | | | | | | | | | | | | | |
| I | 14 | 2 | 4 | 1 | 7 | | 5 | 1 | 1 | 7 | | 4 | 1 | 0 | 9 | |
| II | 64 | 4 | 11 | 14 | 35 | $P=0.036$ | 9 | 9 | 14 | 32 | $P=0.08$ | 5 | 8 | 18 | 33 | $P=0.087$ |
| III | 30 | 2 | 3 | 16 | 9 | | 4 | 8 | 12 | 6 | | 3 | 9 | 7 | 11 | |

ND, not determined; NI, not informative; LOH, loss of heterozygosity; *P*, overall *P*-value.

**Table III** LOH in three *BRCA2* marker regions in invasive ductal carcinomas ($n=108$) correlated with patient's age and histopathological findings

| | | *Chromosome 13q* | | | | | | | | | | | | | | |
|---|---|---|---|---|---|---|---|---|---|---|---|---|---|---|---|---|
| | | *D13S260* | | | | | *D13S267* | | | | | *D13S171* | | | | |
| *Criteria* | n=108 | *ND* | *NI* | *LOH* | *No LOH* | P | *ND* | *NI* | *LOH* | *No LOH* | P | *ND* | *NI* | *LOH* | *NO LOH* | P |
| Age | | | | | | | | | | | | | | | | |
| <50 | 33 | 2 | 3 | 6 | 22 | | 2 | 7 | 7 | 17 | | 18 | 6 | 1 | 8 | |
| ⩾50 | 75 | 22 | 11 | 15 | 27 | $P=0.31$ | 13 | 17 | 17 | 28 | $P=0.65$ | 53 | 9 | 7 | 6 | $P=0.074$ |
| Tumour | | | | | | | | | | | | | | | | |
| T1 | 57 | 12 | 6 | 10 | 29 | | 7 | 14 | 12 | 25 | | 38 | 9 | 3 | 7 | |
| T2 | 40 | 10 | 7 | 9 | 14 | $P=0.50$ | 6 | 8 | 9 | 16 | $P=0.85$ | 27 | 4 | 4 | 5 | $P=0.80$ |
| T3/4 | 11 | 2 | 1 | 2 | 6 | | 2 | 2 | 3 | 4 | | 6 | 2 | 1 | 2 | |
| Nodes | | | | | | | | | | | | | | | | |
| N0 | 54 | 8 | 7 | 13 | 26 | | 7 | 10 | 13 | 24 | | 32 | 9 | 5 | 8 | |
| N1/2 | 54 | 16 | 7 | 8 | 23 | $P=0.67$ | 8 | 14 | 11 | 21 | $P=0.85$ | 39 | 6 | 3 | 6 | $P=1.0$ |
| Grade | | | | | | | | | | | | | | | | |
| I | 14 | 1 | 2 | 3 | 8 | | 3 | 3 | 0 | 8 | | 11 | 2 | 0 | 1 | |
| II | 64 | 17 | 8 | 10 | 29 | $P=0.51$ | 8 | 16 | 14 | 26 | $P=0.055$ | 41 | 10 | 4 | 9 | $P=0.495$ |
| III | 30 | 6 | 4 | 8 | 12 | | 4 | 5 | 10 | 11 | | 19 | 3 | 4 | 4 | |

ND, not determined; NI, not informative; LOH, loss of heterozygosity; *P*, overall *P*-value.

*vs* G3 $P=0.005$; THRA1: overall $P=0.008$, G1 *vs* G3 $P=0.05$, G2 *vs* G3 $P=0.011$), but not with age and lymph node status. For *BRCA2* markers (Table I) losses could be detected in IDC in 30–31%, in ILC in 17–39%, in other invasive breast tumours in 0–33% of cases, but not in benign breast tumours. Only losses of *BRCA2* marker D13S267 in IDC (Table III) correlated significantly with higher grade (overall $P=0.055$, G1 *vs* G3 $P=0.026$). There was no correlation with age, tumour size, and lymph node status. Owing to low numbers, losses of *BRCA1* and *BRCA2* in ILC and other subgroups were not analysed by statistical tests.

To improve genetic information about the individual tumours, losses of all six markers on both chromosomes were combined (Table IV) and analysed in IDC and ILC. In IDC and ILC, cases with exclusive losses of *BRCA1* were twice as abundant as those of exclusive losses of *BRCA2*. In IDC for exclusive losses of *BRCA1* statistical significances compared with the group without losses were seen for tumour size (overall $P=0.074$, T1 *vs* T2 $P=0.01$), for tumour grade (overall $P=0.02$, G1 *vs* G3 $P=0.018$), but not for age and lymph node metastases. Exclusive losses of *BRCA2* did not correlate significantly with any of the parameters analysed. Combination of losses of *BRCA1* and *BRCA2* revealed significant relationships for tumour size (overall $P=0.04$, T1 *vs* T2 $P=0.04$) and tumour grade (overall $P=0.01$, G1 *vs* G3 $P=0.03$, G2 *vs* G3 $P=0.016$) but not for age and lymph node metastases.

For all tumour samples data for steroid hormone receptor (SHR) expression were available. 65% of the tumours were PgR, 63% ER positive. In none of the histological subgroups did ER expression correlate significantly with loss of *BRCA1* or *BRCA2*. For PgR expression, there was a significant

**Table IV** Number of LOH at *BRCA1* or/and *BRCA2* loci of invasive ductal ($n = 108$) and invasive lobular ($n = 19$) carcinoma correlated with patient's age and histopathological findings ($n$ total = 127)

| Criteria | *IDC* *108* | *ILC* *19* | *Total* *127* | *Not informative* *IDC* *(n=6)* | *ILC* *0* | *No LOH* *IDC* *(n=41)* | *ILC* *(n=8)* | *LOH at* BRCA1 *IDC* *(n=21)* | *ILC* *(n=5)* | *LOH at* BRCA2 *IDC* *(n=12)* | *ILC* *(n=2)* | *LOH at two chromosomes* *IDC* *(n=23)* | *ILC* *(n=4)* |
|---|---|---|---|---|---|---|---|---|---|---|---|---|---|
| Age | | | | | | | | | | | | | |
| <50 | 33 | 6 | 39 | 1 | 0 | 13 | 3 | 7 | 1 | 2 | 0 | 7 | 2 |
| ≥50 | 75 | 13 | 86 | 5 | 0 | 28 | 5 | 14 | 4 | 10 | 2 | 16 | 2 |
| Tumour | | | | | | | | | | | | | |
| T1 | 57 | 9 | 66 | 2 | 0 | 26 | 4 | 7 | 2 | 8 | 1 | 9 | 2 |
| T2 | 40 | 8 | 48 | 3 | 0 | 9 | 2 | 13 | 3 | 4 | 1 | 11 | 2 |
| T3/4 | 11 | 2 | 13 | 1 | 0 | 6 | 1 | 1 | 0 | 0 | 0 | 3 | 0 |
| Nodes | | | | | | | | | | | | | |
| N0 | 54 | 7 | 61 | 4 | 0 | 20 | 4 | 9 | 1 | 4 | 1 | 16 | 1 |
| N1/2 | 54 | 12 | 66 | 2 | 0 | 21 | 4 | 12 | 4 | 8 | 1 | 7 | 3 |
| Grade | | | | | | | | | | | | | |
| I | 14 | | 14 | 1 | | 7 | | 0 | | 1 | | 1 | |
| II | 64 | | 64 | 2 | | 28 | | 13 | | 9 | | 11 | |
| III | 30 | | 30 | 3 | | 6 | | 8 | | 2 | | 11 | |
| X | | 19 | 19 | | 0 | | 8 | | 5 | | 2 | | 4 |

inverse correlation in IDC with all three *BRCA1* markers, but none with the three *BRCA2* markers. Combining LOH data of both loci and comparing them with SHR expression showed significant correlations for PgR, but not for ER.

### Discussion

Breast cancer occurs in hereditary and sporadic forms and shows clinical and genetic heterogeneity. Clinically, the onset of breast cancer at an early age, an excess of bilaterality and patterns of multiple primary cancers such as combinations of breast and ovarian cancers point to a hereditary breast–ovarian cancer syndrome (Lynch *et al.*, 1994). In 1990 an autosomal dominant susceptibility gene for breast and ovarian cancer, *BRCA1*, was assigned to chromosome 17q21 by multipoint genetic linkage (Hall *et al.*, 1990 Albertsen *et al.*, 1994) and was later cloned and sequenced (Miki *et al.*, 1994). Observations of LOH for polymorphic markers widely spaced along chromosome 17q (Futreal *et al.*, 1992; Hall *et al.*, 1992; Easton *et al.*, 1993; Knyazev *et al.*, 1993; Kirchweger *et al.*, 1994; Lalle *et al.*, 1994) have led to the hypothesis that *BRCA1* is a tumour-suppressor gene. According to Knudson's model of a tumour-suppressor gene, functional loss of both alleles in breast or ovarian tissues is necessary for malignant transformation to occur. In a familial background a germline mutation is the first step in gene inactivation; for sporadic breast cancer a somatic mutation is supposed to happen. A total of 38 distinct mutations were found among 63 mutations identified through a complete screening of the *BRCA1* gene in 372 patients from high-risk families (Shattuck-Eidens *et al.*, 1995). Up to the present time, in sporadic breast cancer no *BRCA1* mutations have been found, even though somatic mutations could be detected in the coding regions of *BRCA1* in sporadic ovarian tumours (Hosking *et al.*, 1995; Merajver *et al.*, 1995). However, it is thought that the *BRCA1* gene is important in the aetiology of sporadic breast cancer. Indirectly, this hypothesis is supported by a report (Thompson *et al.*, 1995) that transcription of *BRCA1* mRNA varies through progression of breast cancer. Normal breast tissue expresses *BRCA1* mRNA at higher levels than ductal carcinoma *in situ* (DCIS) or IDC. In culture, experimental inhibition of *BRCA1* mRNA expression with antisense oligonucleotides accelerated growth of normal and malignant mammary cells. These studies imply that *BRCA1* may function as a negative regulator of mammary epithelial cell growth whose function is altered either through mutation or LOH.

Although routine *BRCA1* sequencing for point mutations is not practicable technically, the detection of truncated proteins encoded by *BRCA1* mutations is under investigation (Hogervorst *et al.*, 1995). In addition, the sequence of *BRCA2* has been published only very recently (Wooster *et al.*, 1995). For a clinical setting, detection of LOH in various genetic markers of *BRCA1* and *BRCA2* in tumours may therefore be the most appropriate starting point to gain information about tumour biology or individual prognosis. In this study, the routine application of PCR and fluorescent DNA technology for detection of LOH was therefore validated and tested. Microsatellite polymorphisms detecting differences in STR sequences were amplified by fluorescent PCR and analysed in an automated DNA sequencer (Liu *et al.*, 1993; Niederacher *et al.*, 1996). The combination of both methods offers several advantages compared with other staining methods and autoradiography:

(1) It is much faster than Southern blot or radioactive PCR. Separation and direct quantification of PCR products can be performed automatically and requires approximately 3 h without additional staining steps. This allows quantification of as many as 40 individual samples simultaneously and 2–3 gels per day to be run on a routine basis.

(2) Fluorescent-labelled primers and PCR products can be stored, as labelling for both primers and products is stable for several months at −20°C.

(3) The enhanced sensitivity of the fluorescent detection method required 25–30 PCR cycles only to achieve detectable results. Linearity of fluorescence detection covers a much wider range than scanning of autoradiograms or ethidium bromide- or silver-stained gels, resulting in improved quality of data. Therefore, this approach is particularly suitable for the analysis of large series of samples and routine clinical use.

Various groups have analysed LOH at chromosomal region 17q close to the *BRCA1* locus, for example, Futreal *et al.* (1992; THRA1, D17S579), Knyazev *et al.* (1993; THH-59), Cropp *et al.* (1993; 19 polymorphic markers) or Kirchweger *et al.* (1994; AFM155xd12, AFM234td2), but very few studies used the intragenic markers D17S855 or D17S1323 (Futreal *et al.*, 1994; Albertsen *et al.*, 1994). These studies did not intend primarily to use the detection of LOH for clinical purposes, but to focus on the localisation of potential breast cancer candidate genes. Therefore, the number of cases analysed were small (14–55) and correlations with clinical parameters were not extensive. In this study, we intended to obtain clinical information from the detection of LOH of *BRCA1*. For LOH of the *BRCA1* region three different markers, one intragenic (D17S855) and two extragenic markers (THRA1, D17S579), were tested. In IDC, overall rates of LOH varied from 32% to 37% without significant differences between intragenic and extragenic markers. For ILC and other types of invasive breast carcinoma these rates extend from 21–28 to 41–43%. For

ILC this revealed a difference between the intragenic D17S855 and the extragenic THRA1 and D17S579 markers; for other types of invasive breast carcinoma the data were too few in number for statistical analysis. Other studies (Futreal *et al.*, 1992; Knyazev *et al.*, 1993; Kirchweger *et al.*, 1994) have reported 29–79% of LOH, but no differences between tumour types have been stated. This should be taken into consideration when comparing LOH data from different studies. For example for IDC, our data are similar to those by the Kirchweger group, but the data for ILC vary significantly. This implies that: (1) the genetic basis of tumorigenesis varies between different types of breast tumours and (2) the use of a single extragenic marker to characterise LOH of a specific tumour-suppressor gene is not sufficient to obtain clinical information. In hereditary breast cancer (Jacquemier *et al.*, 1995), IDC is by far the most abundant tumour type (below age 40: 100%) and 100% of these tumours are histological grade 3. Grade 3 indicates poor prognosis and implies that *BRCA1*-associated breast cancer is worse than sporadic breast cancer. Here, in sporadic IDC, LOH of *BRCA1* markers correlated with larger tumours and likewise higher grade. Survival data are not available at the moment, but will show in the future whether LOH of *BRCA1* markers independently indicates decreased survival.

LOH data for *BRCA2* in breast tumours are scanty. Andersen *et al.* (1992) and Deng *et al.* (1994) found LOH in 20–46% of sporadic breast tumours, Collins *et al.* (1995) in 88% of familial breast cancers. Anderson *et al.* (1992) differentiated varieties of histological types and stated that LOH of *BRCA2* markers was present only in IDC. In our study, we could detect LOH of *BRCA2* markers not only in IDC (30%), but also in 14–38% in ILC and other types of invasive breast carcinoma. Nonetheless, these LOH rates in sporadic breast cancers were far lower than the rates in breast cancer families (Collins *et al.*, 1995). As stated by Andersen *et al.* (1992) none of the classical parameters correlated with LOH. These findings and the fact that LOH of *BRCA2* was not as abundant as LOH of *BRCA1* indicate that loss of this chromosomal region happens later in the cascade of genetic events than the loss of the 17q region.

Up to now, a comparison of LOH of both chromosomal regions, *BRCA1* and *BRCA2*, in the same study population has not been published. For all histological subgroups analysed, LOH of *BRCA1* was a more abundant phenomenon than LOH of *BRCA2*. This could be explained by the complexity of chromosome 17 with multiple potential targets of LOH (e.g. *TP53*, *HIC1*, *BRCA1*, *NME1*, *MDC*, prohibitin) and overlapping regions of allelic losses. In addition deletion patterns were often consistent with the loss of a large portion of a chromosomal arm or even with the complete loss of chromosome 17 (Kirchweger *et al.*, 1994; Devilee and Cornelisse, 1994; Niederacher *et al.*, 1996). Exclusive LOH of *BRCA1*, as well as combined losses of *BRCA1* and *BRCA2*, always correlated significantly with higher grade, as seen in familial histopathology (Jacquemier *et al.*, 1995). Tumour size, indicating rapid cellular proliferation and division, seemed to be another variable, that might correlate with alteration of *BRCA1* and *BRCA2*. Genetic analyses by linkage and LOH at these specific chromosomal regions implicates the presence of two tumour-suppressor genes. However, it may be possible for LOH of these genes to be randomly acquired and irrelevant to tumour development. Other authors (Chen *et al.*, 1992) have demonstrated an overall 4% background incidence of LOH calculated by LOH analysis of 12 randomly chosen chromosomal regions (Chen *et al.*, 1992). Devilee and Cornelisse (1994) have compiled allelotype data from over 30 studies, comprising more than 1000 sporadic breast tumour specimens. An incidence of LOH below 10% is expected for LOH events unimportant for breast tumour development, probably resulting from genetic instability associated with tumour development and progression. Incidences of LOH of *BRCA1* and *BRCA2* in our study were significantly greater than the arbitrary cut-off defined in the compilation study (Devillee and Cornelisse, 1994), indicating a possible role for *BRCA1* and *BRCA2* in sporadic breast cancer. Whether LOH of these genes is one of the key steps in multistep carcinogenesis of sporadic breast cancer and whether LOH analysis results in more relevant prognostic information has to be verified in a larger study group including earlier stages of sporadic breast cancer and clinical follow-up. However, the LOH analysis of *BRCA1* and *BRCA2* by PCR and fluorescent DNA technology proved to be feasible in the routine setting.

**Acknowledgements**

This work was supported in part by grant Be 1215/6-1 from the Deutsche Forschungsgemeinschaft, Bonn, Germany. HX An was supported by a research fellowship from the Alexander von Humboldt foundation, Bonn, Germany. The authors would like to thank the staff of the operating room and the technicians of the laboratory of pathomorphology, Frauenklinik, Heinrich-Heine Universität, Germany, for the recruitment of the tumours, their expert technical assistance and continued support.

### References

ALBERTSEN HM, SMITH SA, MAZOYER S, FUJIMOTO E, STEVENS J, WILLIAMS B, RODRIGUEZ P, CROPP CS, SLIJEPCEVIC P, CARLSON M, ROBERTSON M, BRADLEY P, LAWRENCE E, HARRINGTON T, MEI SHENG Z, HOOPES R, STERNBERG N, BROTHMAN A, CALLAHAN R, PONDER BAJ AND WHITE R. (1994). A physical map and candidate genes in the BRCA1 region on chromosome 17q12-21. *Nature Genet.*, **7**, 472–479.

ANDERSEN TI, GAUSTAD A, OTTESTAD L, FARRANTS GW, NESLAND JM, TVEIT KM AND BÖRRESEN AL. (1992). Genetic alterations of the tumour suppressor gene regions 3p, 11p, 13q, 17p, and 17q in human breast carcinomas. *Genes Chrom. Cancer*, **4**, 113–121.

BECKMANN MW, TUTSCHEK B, KRÜGER KH, NIEDERACHER D, RISSE BC, RUPPERT C, SCHNÜRCH HG AND BENDER H. (1993). Comparison of the biochemical and immunohistochemical detection of the epidermal growth factor receptor (EGF-R) in breast tumor samples. *Int. J. Oncol.*, **3**, 389–397.

BLACK DM. (1994). The genetics of breast cancer. *Eur. J. Cancer*, **30A**, 1957–1961.

BOYD J. (1995). BRCA1: more than a hiriditary breast cancer gene? *Nature Genet.*, **9**, 335–336.

CHEN LC, KURISU W, LJUNG BM, GOLDMAN ES, MOORE II D AND SMITH HS. (1992). Heterogeneity for allelic loss in human breast cancer. *J. Natl Cancer Inst.*, **84**, 506–510.

COLLINS N, McMANUS R, WOOSTER R, MANGION J, SEAL S, LAKHANI SR, ORMISTON W, DALY PA, FORD D, EASTON DF AND STRATTON MR. (1995). Consistent loss of the wild type allele in breast cancers from a family linked to the BRCA2 gene on chromosome 13q12-13. *Oncogene*, **10**, 1673–1675.

CROPP CS, CHAMPEME MH, LIDEREAU R AND CALLAHAN R. (1993). Identification of three regions on chromosome 17q in primary human breast carcinomas which are frequently deleted. *Cancer Res.*, **53**, 5617–5619.

DENG G, CHEN LC, SCHOTT DR, THOR A, BHARGAVA V, LJUNG BM, CHEW K AND SMITH HS. (1994). Loss of heterozygosity and p53 gene mutations in breast cancer. *Cancer Res.*, **54**, 499–505.

DEVILEE P AND CORNELISSE CJ. (1994). Somatic genetic changes in human breast cancer. *Biochim. Biophys. Acta*, **1198**, 113–130.

EASTON DF, BISHOP DT, FORD D, CROCKFORD GP AND THE BREAST CANCER LINKAGE CONSORTIUM. (1993). Genetic linkage analysis in familial and ovarian cancer: results in 214 families. *Am. J. Hum. Genet.*, **52**, 678–701.

EL-ASHRY D AND LIPPMAN ME. (1994). Molecular Biology of breast carcinoma. *World J. Surg.*, **18**, 12–20.

FORD D, EASTON DF, BISHOP DT, NAROD SA, GOLDGAR DE AND THE BREAST CANCER LINKAGE CONSORTIUM. (1994). Risks of cancer in BRCA1-mutation carriers. *Lancet*, **343**, 692–695.

FUTREAL PA, SÖDERQUIST P, MARKS JR, IGLEHART JD, COCHRAN C, BARRETT JC AND WISEMAN R. (1992). Detection of frequent allelic loss on proximal chromosome 17q in sporadic breast carcinoma using microsatellite length polymorphismus. *Cancer Res.*, **52,** 2624–2627.

FUTREAL PA, LIU Q, SHATTUCK-EIDENS D, COCHRAN C, HARSHMAN K, TAVTIGIAN S, BENNETT LM, HAUGEN-STRANO A, SWENSEN J, MIKI Y, EDDINGTON K, MCCLURE M, FRYE C, WEAVER-FELDHAUS J, DING W, GHOLAMI Z, SÖDERQUIST P, TERRY L, JHANWAR S, BERCHUCK A, IGLEHART JD, MARKS J, BALLINGER DG, BARRETT JC, SKOLNICK MH, KAMB A AND WISEMAN R. (1994). BRCA1 mutations in primary breast cancer and ovarian carcinomas. *Science*, **266,** 120–122.

HALL JM, LEE MK, NEWMAN B, MORROW JE, ANDERSON LA, HUEY B AND KING MC. (1990). Linkage of early-onset familial breast cancer to chromosome 17q21. *Science*, **250,** 1684–1689.

HALL JM, FRIEDMAN L, GUNTHER C, LEE MK, WEBER JL, BLACK DM AND KING MC. (1992). Closing in on a breast cancer gene on chromosome 17q. *Am. J. Hum. Genet.*, **50,** 1235–1242.

HOGERVORST FBL, CORNELIS RS, BOUT M, VAN VLIET M, OSTERWIJK JC, OLMER R, BAKKER B, KLIJN JGM, VASEN HFA, MEIJERS-HEIJBOER H, MENKO FH, CORNELISSE CJ, DEN DUNNEN JT, DEVILEE P AND VAN OMMEN GJB. (1995). Rapid detection of BRCA1 mutations by the protein truncation test. *Nature Genet.*, **10,** 208–212.

HOSKING L, TROWSDALE J, NICOLAI H, SOLOMON E, FOULKES W, STAMP G, SIGNER E AND JEFFREYS A. (1995). A somatic BRCA1 mutation in an ovarian tumour. *Nature Genet.*, **9,** 343–344.

JACQUEMIER J, EISINGER F, BIRNBAUM D AND SOBOL H. (1995). Histoprognostic grade in BRCA1-associated breast cancer. *Lancet*, **345,** 1503.

KIRCHWEGER R, ZEILINGER R, SCHNEEBERGER C, SPEISER P, LOUASON G AND THEILLET G. (1994). Patterns of allele losses suggest the existence of five distinct regions of LOH on chromosome 17 in breast cancer. *Int. J. Cancer*, **56,** 193–199.

KNYAZEV PG, IMYANITOV EN, CHERNITSA OI AND NIKIFOROVA IF. (1993). Loss of heterozygosity at 17p is associated with Her-2 amplification and lack of nodal involvement in breast cancer. *Int. J. Cancer*, **53,** 11–16.

LALLE P, DE LATOUR M, RIO P AND BIGNON YJ. (1994). Detection of allelic losses on 17q12-q21 chromosomal region in benign lesions and malignant tumors occurring in a familial context. *Oncogene*, **9,** 437–442.

LIU ET, HE M AND RAJGOPAL U. (1993). Differential polymerase chain reaction in the analysis of gene dosage. *Semin. Cancer Biol.*, **4,** 47–58.

LUBIN MB, ELASHOFF JD, WANG SJ, ROTTER JI AND TARADA H. (1991). Precise gene dosage determination by polymerase chain reaction: theory, methodology, and statistical approach. *Mol. Cell. Probes*, **5,** 307–317.

LYNCH HT, LYNCH J, CONWAY T, WATSON P, FEUNTEUN J, LENOIR G, NAROD S AND FITZGIBBONS R. (1994). Heriditary breast cancer and family cancer syndroms. *World J. Surg.*, **18,** 21–31.

MERAJVER SD, PHAM TM, CADUFF RF, CHEN M, POY EL, COONEY KA, WEBER BL, COLLINS FS, JOHNSTON C AND FRANK TS. (1995). Somatic mutations in the BRCA1 gene in sporadic ovarian tumours. *Nature Genet.*, **9,** 439–443.

MIKI Y, SWENSEN J, SHATTUCK-EIDENS D, FUTREAL PA, HARSHMAN K, TAVTIGIAN S, LIU Q, COCHRAN C, BENNETT LM, DING W, BELL R, ROSENTHAL J, HUSSEY C, TRAN T, MCCLURE M, FRYE C, HATTIER T, PHELPS R, HAUGEN-STRANO A, KATCHER H, YAKUMO K, GHOLAMI Z, SHAFFER D, STONE S, BAYER S, WRAY C, BODGEN R, DAYANANTH P, WARD J, TONIN P, NAROD S, BRISTOW PK, NORRIS FH, HELVERING L, MORRISSON P, ROSTECK P, LAI M, BARRETT JC, LEWIS C, NEUHAUSEN S, CANNON-ALBRIGHT L, GOLDGAR D, WISEMAN R, KAMB A AND SKOLNICK MH. (1994). A strong candidate for the breast and ovarian cancer susceptibility gene BRCA1. *Science*, **266,** 66–71.

NIEDERACHER D, AN HX, PICARD F, VAN ROEYEN CNC, MOSNY DS, SCHNÜRCH HG, BENDER HG AND BECKMAN MW. (1995). Frequent allelic loss of chromosome 17 in sporadic breast cancer detected by fluorescent labelled microsatellite analysis and automated DNA technology. *Cancer Res.*, (submitted).

SAVITSKY K, BAR-SHIRA A, GILAD S, ROTMAN G, ZIV Y, VANAGAITE L, TAGLE DA, SMITH S, UZIEL T, SFEZ S, ASHKENAZI M, PECKER I, FRYDMAN M, HARNIK R, PATANJALE SR, SIMMONS A, CLINES GA, SARTIEL A, GATTI RA, CHESSA L, SANAL O, LAVIN MF, JASPERS NGJ, TAYLOR AMR, ARLETT CF, MIKI T, WEISSMAN SM, LOVETT M, COLLINS FS AND SHILOH Y. (1995). A single ataxia telangiectasia gene with a product similar to PI-2 kinase. *Science*, **286,** 1749–1753.

SATO T, TANIGAMI A, YAMAKAWA K, AKIYAMA F, KASUMI F, SAKAMOTO G AND NAKAMURA Y. (1990). Allelotype of breast cancer: cumulative allele losses promote tumor progression in primary breast cancer. *Cancer Res.*, **50,** 7184–7189.

SHATTUCK-EIDENS D, MCCLURE M, SIMARD J, LABRIE F, NAROD S, COUCH F, HOSKINS K, WEBER B, CASTILLA L, ERDOS M, BRODY L, FRIEDMAN L, OSTERMEYER E, SZABO C, KING MC, JHANWAR S, OFFIT K, NORTON L, GILEWSKI T, LUBIN M, OSBORNE M, BLACK D, BOYD M, STEEL M, INGLES S, HAILE R, LINDBLOM A, OLSSON H, BORG A, BISHOP T, SOLOMON E, RADICE P, SPATTI G, GAYTHER S, PONDER B, WARREN W, STRATTON M, LIU Q, FUJIMURA F, LEWIS C, SKOLNICK MH AND GOLDGAR DE. (1995). A collaborative survey of 80 mutations in the BRCA1 breast and ovarian cancer susceptibility gene. *JAMA*, **273,** 535–541.

SHIBATA D. (1994). Extraction of DNA from paraffin-embedded tissue for analysis by polymerase chain reaction: new tricks from an old friend. *Hum. Pathol.*, **25,** 561–563.

STRAUSS WM. (1994). Unit 2.2: Preparation of genomic DNA from mammalian tissue. In: *Current Protocols in Molecular Biology*. Ausubel FM, Brent R, Kingston RE, Moore DD, Seidman JG, Smith JA and Struhl K, eds. Vol. 1, Chichester: John Wiley.

THOMPSON ME, JENSEN RA, OBERMILLER PS, PAGE DL AND HOLT JT. (1995). Decreased expression of BRCA1 accelerates growth and is often present during sporadic breast cancer progression. *Nature Genet.*, **9,** 444–450.

WOOSTER R, NEUHAUSEN SL, MANGION J, QUIRK Y, FORD D, COLLINS N, NGUYEN K, SEAL S, TRAN T, AVERILL D, FIELDS P, MARSHALL G, NAROD S, LENOIR GM, LYNCH H, FEUNTEUN J, DEVILEE P, CORNELISSE CJ, MENKO FH, DALY PA, ORMISTON W, MCMANUS R, PYE C, LEWIS CM, CANNON-ALBRIGHT LA, PETO J, PONDER BAJ, SKOLNICK MH, EASTON DF, GOLDGAR DE AND STRATTON MR. (1994). Localization of a breast cancer susceptibility gene, BRCA2, to chromosome 13q12–13. *Science*, **265,** 2088–2090.

WOOSTER R, BIGNELL G, LANCASTER J, SWIFT S, SEAL S, MANGION J, COLLINS N, GREGORY S, GUMBS C, MICKLEM G, BARFOOT R, HAMOUDI R, PANTEL S, RICE C, BIGGS P, HASHIM Y, SMITH A, CONNOR F, ARASON A, GUDMUNDSSON J, FICENEC D, KELSELL D, FORD D, TONIN P, BISHOP DT, SPURR NK, PONDER BAJ, EELES R, PETO J, DEVILEE P, CORNELISSE C, LYNCH H, NAROD S, LENOIR G, EGILSSON V, BARKADOTTIR RB, EASTON DF, BENTLEY DR, FUTREAL PA, ASHWORTH A AND STRATTON MR. (1995). Identification of the breast cancer gene BRCA2. *Nature*, **378,** 789–792.